# Are We Doing More Than We Know? Possible Mechanisms of Response to Music Therapy

**DOI:** 10.3389/fmed.2018.00255

**Published:** 2018-09-11

**Authors:** Amy Clements-Cortes, Lee Bartel

**Affiliations:** ^1^Faculty of Music, University of Toronto, Toronto, ON, Canada; ^2^Wilfrid Laurier University, Waterloo, ON, Canada

**Keywords:** music therapy, neural rhythm, mechanisms, older adults, rhythmic sensory stimulation

## Abstract

Due to advances in medical knowledge the population of older adults struggling with issues of aging like Alzheimer's disease (AD), Parkinson's disease (PD), and stroke is growing. There is a need for therapeutic interventions to provide adaptive strategies to sustain quality of life, decrease neurologic impairment, and maintain or slow cognitive decline and function due to degenerative neurologic diseases. Musical interventions with adults with cognitive impairments have received increased attention over the past few years, such as the value of personalized music listening in the iPod project for AD (1); music as a tool to decrease agitation and anxiety in dementia (2); and music to aid in episodic memory (3); Rhythmic Auditory Stimulation as rehabilitation for PD (4); and recently the potential of 40 Hz sensory brain stimulation with AD and PD (5, 6). These approaches indicate the expanding scope and efficacy of music therapy and the potential mechanisms involved. This paper explicates a four-level model of mechanisms of music response (7, 8) that may help understand current music therapy approaches and treatments and help focus future research. Each level will be illustrated with research and suggestions for research directions.

## Introduction

“The fact that music is implicated in so many different types of interventions relating to health and wellbeing underscores the belief that being moved or touched by music cannot be held purely as a metaphor, which renders music as mere embellishment of our daily lives” [(9), p. 3]. North and Hargreaves (10) propose 10 reasons why music is beneficial, stating music is: ubiquitous, emotional, engaging, distracting, physical, ambiguous, social, and communicative; and music affects behavior and identities. However, music is not only “music” as culturally perceived cognitive product. It is sound and as such vibration—the compression and decompression of molecules in air or material.

Music experiences exist along a continuum, and we believe there is value in each music and sound experience. The distinctions however between experiences and their value are not clearly defined in the literature (11). The two broad approaches of implementing sound experiences for the geriatric as well as other populations are music therapy and music medicine The distinction between these is sometimes not clear, but centers on the sound that is included, the tools or devices that help facilitate the effects, the inclusion and role of the therapist, and from a research perspective, the mechanisms involved. In music therapy, the therapist establishes a trusting relationship with the client and plays a central role along with the music in facilitating the acquisition of goals in multiple domains such as motor, social and emotions. Music therapy looks at treating the whole person, and is broader than music medicine which can be considered to be more of a prescribed approach to music applications.

This paper uses a four-level model of music response mechanisms (7, 8) that may facilitate deeper understanding of music therapy approaches and treatments and focus future research. Each level will be explained with supporting research and suggestions for research directions.

## A model of response mechanisms

Most existing research on music therapy is evidence-based. This is understandable since as a research focus it is relatively new and the first priority to establish clinical legitimacy is to demonstrate that it is effective. However, the next step in developing approaches and refining techniques (12) is to understand why something works, i.e., what is the underlying mechanism. Craver and Bechtel (13) describe four dimensions of mechanisms: (1) a phenomenal dimension—a mechanism does things; (2) a componential dimension— a mechanism has components; (3) a causal dimension—components interact to make the phenomenon happen, and (4) an organizational dimension—components are organized in space and time. For example, we understand well the mechanism of hearing. The phenomenon involves changing compression and decompression of air molecules into the conscious perception of sound. A highly simplified description of components involved includes the ear drum, the malleus, incus, stapes, and cochlea that transmit the vibration to the cilia; and thus to the auditory nerve to the brain where cognitive processes perceive the electrical signals as sound. The particular organization of these components and how they interact causes vibration to be heard.

The question is, what mechanisms are involved in the effectiveness of music therapy. For example, why does the iPod Project approach work with some people? There are at least four levels or types of mechanisms responsible for the effect of music therapy and music medicine. The most dominant and most obvious is the learned cognitive response. The phenomenon of “our song” or specific music associated with a patriotic or religious feeling is the result of learned response within cultural context. Often people can describe how the association developed and explain why the emotional response may occur. But much music learning is accomplished before and without conscious memory. These are isomorphic and primal. For example, babies hear for months before they are born and are exposed to mother's heartbeat and womb sounds and then in the months following birth continue to learn and respond to music (14, 15). There is no doubt babies learn during these very early months (16–19) even though there can be some dispute in the nature vs. nurture vein about what is inherent, hardwired predisposition and what is learned through experience (20, 21). But clearly they learn—pitch patterns, melodies, rhythms, timbre, emotional expression. Although there is little definitive research because of the difficulty of infant learning research, it can be extrapolated that other associations are learned—rapid heartbeat (or rhythm) with energy, stress, and excitement, high pitches with muscle tension and lower pitches with relaxed states. This learning includes all aspects of musical culture including scalar pitch patterns, tonalities, and all expressive conventions (22). As a result many of our responses to sounds and music seem innate and universal but are in fact learned.

**Figure 1:**
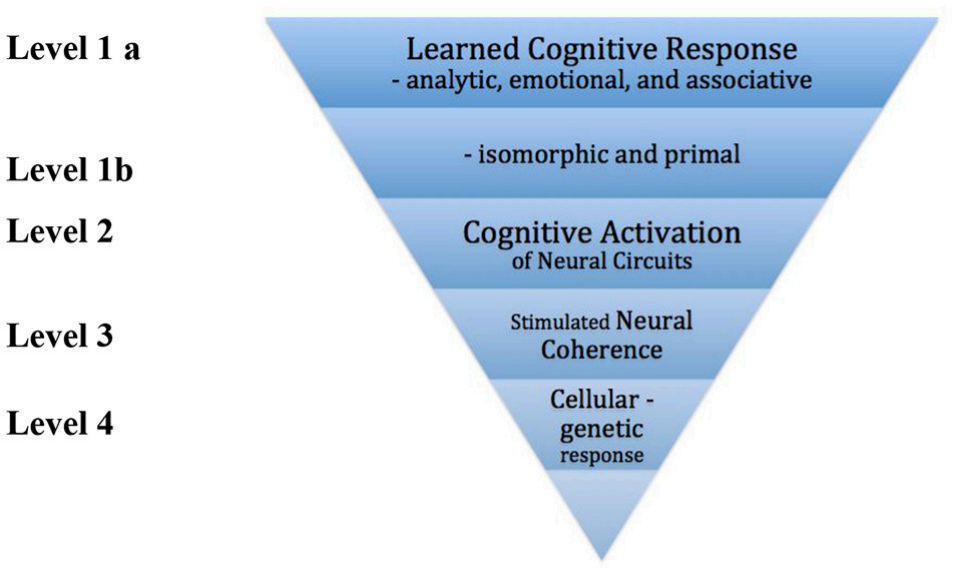
A model of response mechanisms.

Level 2 in our model involves mechanisms of neural circuitry activated by cognitive processes. For example, when a person loses use of language due to a stroke, music with language activates a different circuit in the brain and can thereby rehabilitate language function. This circuit based approach can focus on movement, speech and language, and other cognitive processes such as memory. The practice known as Neurologic Music Therapy is based on neural mechanisms of cognitive processes (23). Level's 3 and 4 presented next are more speculative and for purposes of clarity for music therapy are simplified here. The key concept here is that these responses are not the product of cognitively processing music—they are the response to music at a vibrational rhythmic level. The mechanism of neural or cellular response is complex but there is no doubt that click to neural response is a one-to-one direct relationship (24–27). Level 3 of the model focuses on the mechanism of neural oscillatory coherence—i.e., more neurons firing in synchrony. Rhythmic stimulation of the senses (auditory, visual, tactile) can result in entrainment of neural activity with the potential for several beneficial effects (27–30). Level 4 proposes that there are mechanisms activated by music and sound at the cellular level. This can potentially range from neurons to bone cells to blood cells (31–33) (see Figure 1).

## Level 1a-learned cognitive responses: analytic, emotional, and associative

Engaging in music is nurtured through our emotional experiences with music (33). Bartel (34, 35) suggested physiological and emotional responses to music are created by a collection of learned associations with music, and that these associations are unique to each individual. Associations are formed in childhood and continue to be built upon as we age. They are further reinforced in society through our interactions, the media and cultural events.

By triggering poignant memories that are associated with specific music, music can arouse emotions; and subsequently when a person hears that music, it can activate the associated emotional memory and prompt the event associated emotions. With respect to the geriatric population, understanding this can lead to more intentional inclusion of music therapy interventions for a variety of older adults with varying diagnoses. For example, both emotional and social needs of older adults can be addressed through attendance at a concert, singing and listening to music (36–39). Clements-Cortes (36) found that older adults attending chamber music concerts experienced increased mood and energy and reduced pain.

Three recent singing studies with both cognitively intact and impaired older adults and one which also included caregivers, showed the outcome of this intervention with respect to the emotional, social, and physical dimensions of health (40–42). Singing has also been shown to lower immunoglobulin A and cortisol resulting in feelings of increased levels of relaxation (43, 44) and decreased perception of both acute and chronic pain in single music therapy sessions in hospice patients (45).

### Dementia and AD

Research has demonstrated that music interventions help to reduce agitation, aggression, irritability, restlessness, unwanted verbal behaviors and wandering in persons with AD (46–49). Persons with dementia exhibit these types of behaviors in response to something that is or is not in their environment. For example, a person may be agitated because he/she does not recognize their surroundings and does not feel safe. During these periods engaging the person in making music, activating the mechanism of associated emotion or memory can help change their mood and emotions, while providing a feeling of connection to another and feelings of safety. With respect to another significant concern for persons with AD, Dassa and Amir (50), noted that for persons in middle to late stage AD, singing familiar songs from the past, again activating the mechanism of associated emotion or memory, stimulated conversation and facilitated memory recall.

### iPod project

The 2014 Documentary titled *Alive Inside: The Story of Music and Memory* drove global awareness of the psychosocial and emotional benefits of music for people with dementia (51). Some persons in the movie are described as being awakened when listening to their favorite and preferred music on an iPod. But how, why and when does this happen? There is research pointing to the benefits of music listening for persons with dementia including: decreased agitation (52, 53) and reduced irritability in group music listening experiences (54); however, the documentary does not accurately portray the mechanisms caregivers or healthcare professionals utilize to structure, facilitate, and monitor this music listening. For example, one of the persons in the film “Henry” sings along to his “favorite” music from the past while listening to music on the iPod. He recalls the song lyrics and becomes emotional, responsive to others in his environment and animated. The reason music listening works so well here, is that the person who prepared the music listening opportunity found music that had positive connections and meaning to Henry; and as a result Henry responds progressively. However, because response-triggering associations to music are individually learned, selecting the right music is not an easy task. Just because someone likes a particular artist or style of music, does not mean we can easily pick the right songs for them to have the same response as Henry did. Further, there is issue with putting headphones on a person with dementia and leaving them alone to listen to music, as they cannot tell you to take off the headphones, ask to have the music stop, or be validated if they start to cry or have a negative reaction to the music when a caregiver is not present. Also important to mention is the severity of the dementia and comorbidities. Music is a social experience, and it would be more beneficial to sit and listen to music together when a person has dementia, while engaging them in discussion or movement in order to maximize the potential of this intervention. Taking a mechanism perspective will reduce the tendency to rely on “the power of music” and to see specific music as individually learned “triggers” with unique responses. Effective music listening opportunities can be created when they are carefully planned. Guidelines on preparing these opportunities have been developed by music therapists (55).

## Level 1b—learned responses—isomorphic and primal

Differentiating a response to music that has been learned however early, from a response that may be evolutionarily biological and genetically innate is very difficult, and few explanations have been attempted because research in neuropsychology of musical emotion is in its infancy (56). But it is quite safe to assume that babies, who start to hear months before they are born, learn from sound—their mother's voice, the rhythm of the heartbeat, etc. Since music is based on temporal patterns of vibration plus sequential and simultaneous aspects (57) the earliest learnings or imprints of heart rhythms form the basis of response—fast equals excitement, slow equals calm. This isomorphic (similar in form, shape, or structure) relationship extends as well to louder being stronger and bolder; and quieter being weaker and softer. The Hevner Adjective Circle illustrates how pervasive these primal (from early stages of development) responses are (58). A review of the music therapy examples in the section below will make it evident how pervasive this level of response is to music. Research to control this dimension more specifically might help refine therapeutic use of music.

## Level 2—cognitive activation of neural circuits

Making music is a function that requires auditory, emotional, linguistic, motor, and structural memories (59). Attention, auditory perception, executive function, memory, and motor control are activated when a person makes music (60). Each of these involves brain circuits. For persons who have suffered damage to the hippocampus and parahippocampal regions of the brain (areas associated with memory), research has demonstrated that these areas are activated when a person listens to pleasant music (61). Further, background music is linked to significant improvements in cognitive functioning in older adults including those with AD (62). Neuroscientific research identifying brain circuit activation mechanisms available through music therapy is improving effective implementation of music linked to specific therapeutic outcomes. This approach is known as Neurologic Music Therapy (NMT) (63) or as Music—Supported Therapy (64). The theoretical foundation and research evidence from fMRI studies are detailed in the work of Thaut (65) and Altenmüller (64) and will not be included here.

NMT is defined as the “therapeutic application of music to cognitive, sensory, and motor dysfunctions due to neurologic disease of the human nervous system” (66). NMT uses the perception of auditory structures and patterns in music as specific circuit activators to retrain brain function (67). The techniques are organized into three main areas: sensorimotor training, speech/language training, and cognitive training. The following are three typical applications with older adults.

### An example of sensorimotor training: Parkinson's (PD)

For persons with PD, a common NMT intervention is Rhythmic Auditory Stimulation (RAS). RAS is the application of rhythmical auditory stimuli related to the initiation and facilitation of gait and activities related to gait (68). Dreu et al. (69) concluded there is strong evidence that implementing RAS with gait training leads to improvements in step length and walking velocity. Why does this work? The circuits connecting the cerebellum, basal ganglia, and supplementary motor area (SMA) are what enable a person to initiate and control motor performance; but for the circuit to work, an internal cue is needed (70). With PD, this internal cue is typically missing or impaired (70). RAS is thought to work by providing the person with an external cue: rhythm activates the neural circuit and enhances or replaces the weakened inner cues for the circuit to function.

### An example of speech/language training: AD and non-fluent aphasia

Thaut (71) stated “data suggest that neuronal memory traces built through music may be deeply ingrained and more resilient to neurodegenerative influences (p. 179).” Non-fluent aphasia can be the result of a traumatic brain injury, stroke, dementia, or AD. Musical Speech Stimulation (MUSTIM) is an NMT technique based on a classic language reading teaching strategy known as “cloze procedure” (72). At the simplest level, a therapist would sing the line of a familiar song for the client and leave out the last word, requiring the client to sing or speak it (73). More advanced levels of this technique would involve asking the client a question, practicing common overlearned sentences or to present sentences to the client with potential different outcomes (73). MUSTIM works because brain channels to non-propositional speech often remain intact (73), while propositional language is impaired or disrupted in a person who has non-fluent aphasia. The music therapy mechanism involved is access to “subcortical thalamic speech circuitry” [(73), p. 146].

### A second example of speech/language training: stroke—expressive aphasia

Many persons who suffer from a stroke are left with expressive or Broca's aphasia. One of the most researched and commonly implemented NMT techniques with this population is melodic intonation therapy (MIT). MIT uses “melodic and rhythmic elements of intoning (singing) phrases and words to assist in speech recovery” [(74), p. 140]. Clinical protocols have been established and in 1999, a shorter 6 step structure for clinical efficiency was developed (66). The research related to MIT is encouraging, however the majority of studies often involved small sample sizes (74). A few studies have demonstrated evidence for neuroplasticity prompted by MIT (75–77). MIT is best implemented with clients when there can be multiple sessions in the same week. So what mechanisms are supported or working in MIT? The simple explanation is that sung language uses a different brain channel to access Broca's area and so “singing” speech can function even when direct spoken language access to Broca's area does not. The theoretical explication of this is presented by Schlaug et al. (77, 78). Schlaug et al. (78) noted that hand tapping used in MIT greatly adds to its effectiveness. For example, Poeppel et al. (79) proposed the right hemisphere of the brain is potentially more advanced at handling slowly-modulated signals, whereas the left hemisphere is possibly more perceptive to rapidly-modulated signals. What this suggests is that the constant voicing and slowed pace of articulation of MIT could increase the connection between the words and syllables in singing, and potentially require less effort from the left hemisphere. Further, the sensorimotor network (SMN) might be more easily activated by tapping the left hand as the SMN controls both mouth and hand movements (80) pointing to a pre-mediated condition preparing the brain to organize a motor task.

## Level 3—stimulated neural coherence

Specific frequency bands of neural oscillatory coherence, commonly referred to as brainwaves, have long been thought to be associated with particular brain states. For example, when oscillation in the 0–4 Hz band, known as delta, is dominant, sleep is a probable brain state. It is now thought that a function of neural oscillatory coherence is the routing and gating of information in circuits (81) with oscillations in the 4–12 Hz range related to interactions over distance from one area to another, and that oscillations in the 20–100 Hz (gamma) range are related to local communication. It is clear that external rhythmic stimulation of the brain can drive a greater number of neurons into synchronization. This rhythmic stimulation can be accomplished with sensory means (sound, light, touch) and electric means (e.g., transcranial magnetic stimulation) (82). We use the term “rhythmic” in this context as recurring stimulus that can be a short 2 ms auditory “burst” that occurs once a second (1 Hz) or can occur 10 times a second (10 Hz) or 60 times a second (60 Hz). It could also be a flash of light occurring at these frequencies. Or it could be a “burst” of electrical stimulation. Sound waves at a 100 Hz are still a rhythmic, i.e., periodic, stimulation to the ear by means of compressions of air molecules.

The question for music therapy and music medicine is how rhythmic aspects of sound can function as a neural coherence stimulant. It is at the level of this mechanism that we may be doing more than we know. And, the potential at this level is substantial if we can effectively use sound stimulation to support, enhance, or even rehabilitate dysrhythmias in neural circuits.

In music medicine Vibroacoustic Therapy (VAT) involving sound driven vibrotactile stimulation has been used for many years, but has been studied primarily as a physical muscular stimulant and frequently with effects inexplicable with purely physiological mechanisms. It can now be subsumed into Rhythmic Sensory Simulation (RSS) with its added focus on neural stimulation by auditory, visual, or vibrotactile means. Research investigating RSS in geriatrics is beginning to grow. Below we focus on a few examples with respect to AD and Parkinson's.

### Alzheimer's disease

In a study implementing sound-based RSS quantitative results indicated: improved cognition, clarity, and alertness in persons with AD (5). In the study 40 Hz vibrotactile and auditory stimulation (30 min) were delivered by a vibroacoustic chair twice a week for 3 weeks. The St. Louis University Mental Status test (SLUMS) showed an effect size of 0.58 for each session (total performance gain of about 12%). A 3 year longitudinal case study of a female with AD using two vibroacoustic devices to provide RSS stimulation pointed toward the potential of RSS to assist in the maintenance of cognition over time (83). The participant's Mini Mental State Examination (MMSE) score remained the same from pre- to post- study. Important to note this is a single case which adds support, but also must be considered as such with respect to being weighty evidence. The putative mechanism involved in these effects is an observed reduction in gamma amplitude around 40 Hz in AD patients (84) resulting in weakened cognition and memory circuits and that RSS at 40 Hz drives up this amplitude restoring the function of these circuits.

A study with 5XFAD mice using light-based RSS, is strongly supportive to the efficacy of 40 Hz brain stimulation but possibly with a different mechanism. Iaccarino et al. (27) found 40 Hz light flicker for 60 min for 7 days demonstrated: reduction in amyloid-β of over 50%, increase in microglia activity, increase in blood vessel diameter, reduction in inflammation, and improvement in memory, spatial orientation, and learning. These researchers report the same or greater effect with 40 Hz auditory stimulation and are now doing a human trial using visual, auditory, and vibrotactile RSS (30).

Electric stimulation of the brain for AD is beyond the scope of this paper but positive results are reported by Lozano and Lipsman (85) and Cotelli et al. (86). The relationship between vibrotactile and auditory RSS used in music medicine and electro-stimulation is examined in Bartel et al. (82).

### Parkinson's disease

Explanation of Level 2 of the Mechanism Model above pointed to the effectiveness and method of RAS (57). Parkinson's disease (PD) is more complex, however, than merely a cuing deficit. It is a motor circuit communication problem that is addressed with deep brain stimulation by “silencing” the group of neurons that interferes with the healthy function of the circuit. Inversely the approach of RSS is to boost the oscillatory coherence (electro-potential amplitude) of the primary motor circuit. King et al. (6) demonstrated this in a parallel cross-over design study evaluating the influence of 5 min of vibrotactile and auditory RSS at 30 Hz on functional measures and motor symptoms in persons with PD. Significant improvements were demonstrated for all symptom, functional, and motor control measures, with decreases in tremor and rigidity and increases in step length and speed on the grooved pegboard task. San Vincente et al. (87) found similar effectiveness with 40 Hz and music stimulation.

## Level 4—cellular and genetic

Response to music, sound, and vibration at the cellular and genetic level is relatively unexplored in research and not a conscious focus for music therapists. But, there are important signs from research that this may warrant more development of clinical applications. We will look specifically at neurogenesis, blood flow, bone cell density, and epigenetics.

Using a rationale of deteriorating neural function in dementias, Koike et al. (31) exposed neural cells (PC12m3) *in vitro* and with nerve growth factor (NGF) to a range of sound waves from 10 to 200 Hz for 30 min. They found that sound from 150 to 200 Hz had hardly any effect on neurite outgrowth but that 10–100 Hz induced significant neurite growth. Most effective was 40 Hz that produced triple the amount of growth than NGF alone. The mechanism here appeared to be the sound activating p38 mitogen—activated protein kinase (MAPK) which has a significant effect on neurite growth. This mechanism may be relevant to the findings of Clements-Cortés et al. (5) and Iccarino (27).

Uryash et al. (88) explored the effect of low amplitude pulses on blood vessels. They found that the vibration activated the endothelium to increase release of nitric oxide which induced dilation of the blood vessel and thereby improved blood flow. Uryash et al. (89) present evidence that 50 Hz vibroacoustic stimulation applied to the upper torso can use the endothelial membrane to transform the sound into mechanical energy at the cellular level. Further, that the 50 Hz stimulation enhances myocardial relaxation, improves left ventricular output, and augments coronary flow. Hoffman and Gill (90) have proposed that low frequency vibration (35 Hz) can induce coronary angiogenesis and thereby improve flow of blood to the heart muscle.

Lau et al. (91) explored the effect of 60 Hz stimulation on bone growth and density. They report that vibration has previously been shown to be anabolic (increase) to bone. They tried to identify the specific mechanism and observe that the effect occurs as a result of a cell's mechanosensing. Zheng et al. (32) looked at the effect of low frequency sound stimulation at various frequencies in the range of 27–113 Hz on blood flow and bone metabolism in frail old men and women. They found that the stimulation decreased total osteocalcin and tartrate-resistant acid phosphatase 5b, which has the effect of reducing bone loss.

The least explored area for the effect of music is the genetic level. In a unique study by Kanduri et al. (92) the epigenetic effect of 2 h of making music by professional musicians on their RNA transcriptome. The found that the music performance experience resulted in an expression change in 73 genes (51 up and 22 down regulated). The effect of this is highly complex but is an indicator of the potential response effect at this level of mechanism. Kanduri et al. (93) also studied the effect of listening to classical music for 20 min. They found that people with high level of musical ability and experience had 97 differentially expressed genes with 75 up-regulated and 22 down regulated. Among the up-regulated genes were ones related to dopamine secretion. Since listening to music is known to reduce stress, epigenetics associated with stress reduction probably apply to music listening more broadly (94).

## Conclusion

Several conclusions can be drawn from this examination of a model of response mechanisms for music in therapy. First, the work of music therapists is complex and potent at many levels. Knowledge of and control at the level of the mechanism is important to the maximum clinical effect of the therapist. Secondly, levels 3 and 4 of our model need to be embraced, studied, and applied by music therapy practitioners. Thirdly, a mechanism perspective is needed in research to advance the field. Fostering collaborations between neuroscientists, neurologists and music therapists is recommended as an avenue to further pursue needed research investigations.

## Author contributions

AC-C and LB both contributed to the writing of the paper. AC-C sketched out the paper and the sections were divided up. LB contributed the information about the proposed mechanism and AC-C and LB found the supporting research. AC-C formatted the paper and all references. LB proof read the paper and tracked editing suggestions. AC-C completed final rewrite. AC-C and LB approved the final copy of the paper submitted.

### Conflict of interest statement

The authors declare that the research was conducted in the absence of any commercial or financial relationships that could be construed as a potential conflict of interest.
